# The Therapeutic Potential of Cannabidiol in the Management of Temporomandibular Disorders and Orofacial Pain

**DOI:** 10.3390/pharmaceutics17030328

**Published:** 2025-03-03

**Authors:** Karolina Walczyńska-Dragon, Anna Kurek-Górecka, Jakub Fiegler-Rudol, Aleksandra Nitecka-Buchta, Stefan Baron

**Affiliations:** 1 Department of Temporomandibular Disorders, Medical University of Silesia in Katowice, 41-800 Zabrze, Poland; aleksandra.nitecka@sum.edu.pl (A.N.-B.); sbaron@sum.edu.pl (S.B.); 2 Department of Microbiology and Immunology, Faculty of Medical Sciences, Medical University of Silesia in Katowice, 41-808 Zabrze, Poland; 3 Student Scientific Society at the Department of Temporomandibular Disorders, Medical University of Silesia in Katowice, 41-800 Zabrze, Poland; s88998@365.sum.edu.pl

**Keywords:** temporomandibular disorders, CBD, cannabis, muscle hyperactivity, inflammation

## Abstract

**Background**: Temporomandibular disorders (TMDs) are a group of conditions affecting the temporomandibular joint (TMJ) and associated muscles, leading to pain, restricted jaw movement, and impaired quality of life. Conventional treatments, including physical therapy, medications, and surgical interventions, have varying degrees of success and potential side effects. Cannabidiol (CBD), a non-psychoactive component of cannabis, has gained attention for its anti-inflammatory, analgesic, and anxiolytic properties. This study explores the potential role of CBD in TMD management. **Methods**: A review of existing literature was conducted (2007–2024), focusing on preclinical and clinical studies assessing the efficacy of CBD in pain modulation, inflammation reduction, and muscle relaxation. Relevant studies were sourced from PubMed, Scopus, and Web of Science databases. Additionally, potential mechanisms of action, including interactions with the endocannabinoid system, were analyzed. **Results**: Studies suggest that CBD exerts analgesic and anti-inflammatory effects by modulating CB1 and CB2 receptors, reducing cytokine release, and influencing neurotransmitter pathways. Preliminary clinical evidence indicates that CBD may alleviate TMD-related pain and muscle tension with minimal adverse effects. However, high-quality randomized controlled trials are limited. **Conclusions**: CBD demonstrates promise as a potential adjunctive treatment for TMD. Further research, including well-designed clinical trials, is necessary to establish its efficacy, optimal dosage, and long-term safety.

## 1. Introduction

### 1.1. Rationale

**Figure 1:**
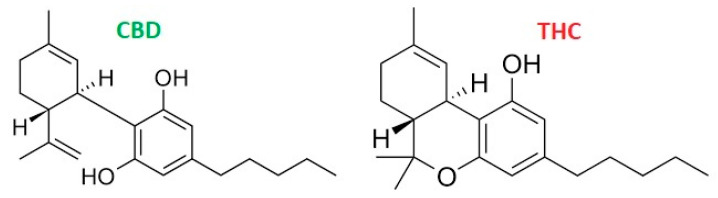
Cannabidiol (CBD) and tetrahydrocannabinol (THC) chemical structures.

Temporomandibular disorders (TMD) comprise various conditions affecting the temporomandibular joint (TMJ), masticatory muscles, and related structures in the head, neck, and shoulder girdle [1,2]. Patients often present with acute or chronic pain, restricted jaw movement, headache (including migraine-like pain), ear or eye discomfort, and muscle overactivity that can radiate to the teeth and cervical region. These symptoms frequently disrupt sleep quality, overall well-being, and psychological health. While standard interventions—such as occlusal splints, physical therapy, behavioral modification of parafunctional habits, and patient education—can effectively relieve many symptoms [3], more severe or persistent cases may involve inflammatory processes (e.g., synovitis, capsulitis, retrodiscitis) that damage TMJ structures through mediators like prostaglandins, leukotrienes, bradykinin, and serotonin [4,5]. Such ongoing inflammation underscores the need for complementary treatment strategies. The endocannabinoid system (ECS), which includes CB1 and CB2 receptors, is pivotal for musculoskeletal homeostasis and pain modulation [6,7,8]. Cannabidiol (CBD), a non-psychoactive component of cannabis, demonstrates promising analgesic and anti-inflammatory effects, indicating its potential to alleviate both muscle hyperactivity and inflammatory pain associated with TMD [6,7,8]. Consequently, this review examines the utility of CBD in managing TMD-related symptoms, such as pain and inflammation, along with other secondary effects. As illustrated in Figure 1, CBD is an organic compound derived from hemp that differs pharmacologically from tetrahydrocannabinol (THC), the primary psychotomimetic agent in cannabis [9,10]. The choice of cannabis strain depends on the intended use and cannabinoid composition. Research has shown CBD to be effective in addressing a wide spectrum of conditions—including epilepsy, Alzheimer’s disease, depression, dystonia, schizophrenia, psychosis, stroke, inflammatory disorders, rheumatoid arthritis, various cancers, nausea, cardiovascular diseases, and diabetic complications [11,12,13,14]. Unlike THC, CBD does not produce psychoactive effects. Numerous studies have verified its anti-inflammatory, analgesic, and muscle-relaxant properties [13,14], prompting growing interest in CBD-based interventions for TMD—particularly when pain results from muscle tension, hyperactivity, or TMJ inflammation.

### 1.2. Objectives

Accordingly, this review aims to critically evaluate the existing literature on the potential efficacy and safety of CBD for TMD, emphasizing its effects on pain reduction, muscle hyperactivity, and inflammation, as well as its possible routes of administration. By synthesizing current evidence, the review seeks to elucidate CBD’s therapeutic potential in TMD treatment and highlight areas in need of further research.

## 2. Materials and Methods

### 2.1. Research Scope and Identification of the Research Area

This review aimed to examine the potential application of CBD in managing TMD, with particular attention to its effects on pain reduction, muscle hyperactivity, and inflammation. At the outset, we formulated our core research question: “How does the use of CBD influence symptom severity, functional outcomes, and patient well-being in TMD?” This inquiry guided the inclusion of articles centered on CBD-based treatments, dosing strategies, and clinical outcomes pertinent to TMD therapy.

### 2.2. Literature Search Strategy

A comprehensive literature search was performed in PubMed, Embase, and Scopus, using both MeSH terms and free-text keywords to capture a broad range of studies on CBD and TMD. Table 1 presents the main search terms employed. We opted to include studies from 2007 to the present, reflecting more recent developments in CBD research. However, a small number of older articles were also considered if they offered foundational theoretical or historical context relevant to TMD pathophysiology or the endocannabinoid system.

### 2.3. Screening and Selection Criteria

A total of 854 records were identified through database searches. After duplicates were removed, the remaining studies underwent title and abstract screening in accordance with the inclusion and exclusion criteria outlined in Table 2. Studies focusing on the analgesic or anti-inflammatory properties of CBD in TMD or orofacial pain were included. In general, studies were included if they (1) investigated cannabidiol (CBD) in the context of temporomandibular disorders (TMD) or orofacial pain, (2) reported clinical or experimental outcomes related to TMD symptom relief or muscle hyperactivity, and (3) were peer-reviewed articles available in full text. Studies were excluded if they (1) did not focus on TMD, (2) lacked specific findings on CBD interventions, or (3) were conference abstracts, letters, or commentaries without empirical or theoretical rigor. 

### 2.4. Full-Text Review and Data Extraction

Articles deemed eligible after the initial screening were retrieved for full-text evaluation by two independent reviewers. If discrepancies arose, a third reviewer provided the final decision. Data extraction focused on study design, participant characteristics, CBD intervention details (e.g., dose, route of administration), and TMD-related outcomes (e.g., pain level, muscle hyperactivity, TMJ function). Notable methodological limitations—such as small sample sizes, short follow-up durations, or lack of standardized outcome measures—were also recorded.

### 2.5. Elaboration of Results and Synthesis

Following a narrative review methodology, we summarized the key findings qualitatively and organized them into thematic areas: (1) CBD administration routes (topical, oral, or injectable), (2) TMD-related outcomes (pain reduction, muscle hyperactivity, jaw function), (3) anti-inflammatory mechanisms (e.g., cytokine modulation), and (4) safety and tolerability (reported adverse events or dosage considerations). The evidence was then synthesized to highlight benefits, challenges, and critical knowledge gaps in using CBD for TMD. Due to the variability in study designs, participant populations, and outcome measures, a formal meta-analysis was not conducted.

### 2.6. Data Extraction

After identifying eligible studies through title and abstract screening, full-text articles were retrieved and independently evaluated by two reviewers. Key data points were systematically extracted from each study, including study design, participant characteristics, details of the CBD intervention (such as dosage, route of administration, and treatment protocols), and relevant outcomes related to TMD—namely pain reduction, muscle hyperactivity, and joint function. When discrepancies arose between reviewers, a third reviewer intervened to reach a consensus, thereby reinforcing the accuracy and consistency of the extracted data.

## 3. Results

### Study Selection

**Figure 2:**
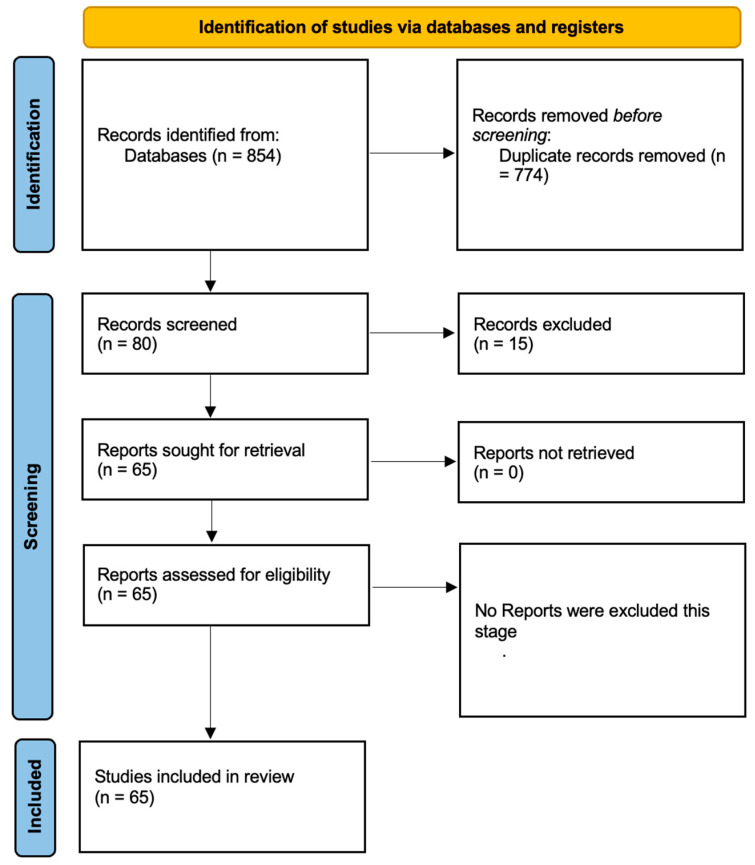
Prisma 2020 flow diagram.

Figure 2 illustrates the study selection process in accordance with PRISMA guidelines [15]. The initial database search across PubMed, Embase, and Scopus yielded 854 unique records after deduplication. Titles and abstracts were then screened against the inclusion and exclusion criteria outlined in Table 2, resulting in a subset of potentially relevant articles that underwent full-text evaluation. Ultimately, 65 articles satisfied the eligibility requirements and were included in this review. 

## 4. Discussion

### 4.1. CBD Properties

CBD has been evaluated by the World Health Organization (WHO), which concluded in November 2017 that it exhibits no potential for abuse or dependence in humans [16]. However, despite moderate abuse and dependence risks—lower than many legal and illegal substances—psychoactive cannabinoids in cannabis (e.g., Δ9-THC) exhibit reinforcing effects, tolerance, and a moderate withdrawal syndrome, non-psychoactive cannabinoids show no intoxicating or rewarding properties, the role of CBD in mitigating THC’s effects remains uncertain, and potent synthetic cannabinoid receptor agonists pose a substantially higher risk of harm [17,18]. 

Furthermore, no public health-related problems have been associated with the use of pure CBD [16]. In January 2018, the World Anti-Doping Agency removed CBD from its prohibited list, thereby allowing athletes to utilize it [19]. Research has identified CBD as beneficial for managing certain medical conditions, such as muscle hyperactivity and chronic pain [20]. Notably, CB1 and CB2 receptors have been found in human fascia and fascial fibroblasts, implying a regulatory role in inflammation and pain within the masticatory muscles [20]. The discovery of cannabinoid receptors in various oral tissues opens up a range of potential approaches to orofacial pain management [20,21]. CBD also demonstrates a strong safety profile in humans, with mostly mild adverse reactions reported, including ataxia, sedation, nausea, headache, and decreased appetite [21]. Although both THC and CBD are cannabinoids, their metabolic pathways differ considerably [22]. THC is primarily converted into 11-hydroxy-THC, an active metabolite contributing to its psychoactive properties, and is then further metabolized into inactive forms (e.g., THC-COOH) excreted via urine and feces [21,22]. Factors such as dose, frequency, and individual enzyme variability influence this process. By contrast, CBD undergoes extensive metabolism but does not yield the same level of psychoactive metabolites. Instead, it is mostly transformed into 7-hydroxy-CBD and other inactive forms, ultimately eliminated in urine and feces [21,22]. Because CBD does not produce a pronounced “high,” it is often chosen by those seeking therapeutic benefits without psychoactive effects. The metabolism of both THC and CBD can be affected by genetics, age, liver function, and concurrent medications—particularly those that inhibit or induce CYP450 enzymes—potentially altering their efficacy and duration [22]. Given its notable therapeutic properties, CBD has attracted considerable scientific attention for various possible applications [20,21,22]. Jakub Mlost et al. [8] suggest that CBD, alone or in combination with other compounds, may exhibit anti-nociceptive effects in several pain-related conditions [8]. Research further indicates that CBD may provide analgesic and anti-inflammatory benefits in inflammatory-induced chronic pain models, including orofacial pain. Both preclinical and clinical studies underscore CBD’s promising anti-nociceptive potential in pain-related disorders [8,20].

#### 4.1.1. CBD Properties in Preclinical Studies

Min K. Lee et al. [23] have demonstrated that the central administration of cannabinoids reduces inflammatory nociception in the temporomandibular joint [23]. Cannabinoids are known to inhibit nociceptive transmission in the spinal cord, and their potency and efficacy to produce antinociception are comparable to that of morphine [23]. The authors conducted the study in rats and used intracisternal injection of aminoalkylindole derivative WIN 55, 212-2 in amount 30mg, which play a role as an agonist of CB1 and CB2 [23]. Their observations suggest that opioid and CB receptors may function together within the same cell or neuronal circuit to produce antinociception, and modulation of one receptor system may lead to alterations in the activity of the other [23,24]. In addition, the administration of cannabinoid receptor agonists is known to produce antinociception and reduce hypersensitivity in neuropathic and inflammatory pain models [25]. Electrophysiological studies have demonstrated that cannabinoid receptor agonists inhibit nociceptive neuronal activity in the spinal trigeminal nucleus caudalis [26].

#### 4.1.2. CBD Properties in Clinical Studies

Several clinical studies have reported on the efficacy of co-administration of CBD and Δ9-THC, typically in doses of 2.5 mg CBD and 2.7 mg Δ9-THC in an oral mucosa spray. Patients who received this treatment reported reduced pain, improved sleep quality, and reduced insomnia and fatigue. However, to the best of our knowledge, there have been no studies conducted on the intraoral administration of pure CBD [27,28,29].

### 4.2. CBD Ways of Administration

Ongoing research continues to investigate various methods of administering CBD in order to maximize its absorption and effectiveness [27,28,29]. Multiple formulations—such as topical gels, oral capsules, or injectable solutions—have been studied to determine their relative advantages and to identify any potential side effects. Table 3 and Table 4 detail the different treatment protocols and dosage regimens of CBD that have been evaluated in both preclinical and clinical settings [23,26,27,30,31]. By examining this evolving body of evidence, researchers aim to clarify how CBD can be applied most effectively for therapeutic purposes, ensuring both optimal pain relief and patient safety [31].

#### 4.2.1. CBD Ways of Administration in Preclinical Studies

Wong et al. demonstrated in preclinical work that the trigeminal ganglion contains CB1 and CB2 receptors innervating the rat masseter muscle [32]. In their study, intramuscular administration of CBD alone (5 mg/mL) lowered mechanical sensitization and raised the mechanical threshold of masseter muscle mechanoreceptors [32]. These findings indicate that injecting CBD could offer analgesic relief for myofascial pain syndrome, notably without inducing neurological side effects [32]. Another rat-based investigation revealed that electroacupuncture exerted anti-inflammatory and antinociceptive effects in TMJ arthritis models. This result suggests that beneficial effects on TMJ might be mediated by CB receptors, thereby underscoring the potential of the endocannabinoid system as a therapeutic pathway for alleviating pain and inflammation in TMD [33]. Together, these observations imply a promising new direction for treating TMJ-related pain. Additional studies show that CBD inhibits NF-κB (nuclear factor kappa-light-chain-enhancer of activated B cells)—a key element in the inflammatory response—and diminishes the production of various interleukins known to promote inflammation [25]. Moreover, CBD modulates or suppresses the synthesis of cytokines, chemokines, and pro-inflammatory growth factors, all of which are involved in the inflammatory process [34,35]. Among these cytokines, Tumor Necrosis Factor, Interleukin-1β (IL-1β), Interleukin-6 (IL-6), and Interferon-gamma have been widely investigated, and their levels consistently declined following CBD treatment, indicating that CBD directly curtails inflammation. In a preclinical study by Vivanco-Estela et al., the authors noted a distinct difference in treatment outcomes based on sex [36]. They found that male rats exhibited greater reductions in orofacial allodynia and hyperalgesia than female rats [36]. Specifically, in females, acute administration of lower and intermediate CBD doses (10 and 50 μg) into the masseter muscle lessened orofacial allodynia; in males, it was the intermediate and higher doses (50 and 100 μg) that had this effect [36]. All three CBD doses reduced orofacial hyperalgesia in both male and female rats, with males showing a stronger response [36]. These data suggest that localized CBD treatment in the masseter muscle may be a promising therapeutic avenue for individuals experiencing orofacial pain [36].

#### 4.2.2. CBD Ways of Administration in Clinical Studies

Over many years, CBD has demonstrated beneficial effects, leading to numerous clinical trials. For instance, a transdermal gel containing CBD eased pain, cold sensations, and itching in individuals with peripheral neuropathy. CBD can be used on its own or in combination with other cannabinoids and using multiple cannabinoids together may provide broader therapeutic advantages [30,37]. In a clinical trial evaluating topical CBD application in adults, Nitecka-Buchta et al. found that CBD might exert analgesic effects in temporomandibular joint disorders. In this double-blind trial, masseter muscle activity decreased, and the condition of the masticatory muscles improved following application of a topical CBD formulation in patients with myofascial pain [30]. Randomized controlled trials (RCTs) have shown that CBD can be administered topically, intramuscularly, or through the intraoral mucosa to alleviate orofacial pain. In their study, Wong et al. reported that an intramuscular injection of CBD alone (5 mg/mL) or a CBD:CBN combination (1:1 mg/mL) could offer analgesic relief for myofascial pain syndrome, without causing neurological side effects [38]. A systematic review by Weerathataphan et al. (2021) similarly underscored the importance of cannabinoids in addressing temporomandibular joint (TMJ) pain, noting that the anti-inflammatory and antinociceptive properties of CBD may be activated via CB receptors. The authors called for further research to confirm these pain-reducing effects in clinical settings and to identify the most effective methods of CBD administration [39]. CBD also functions as an inverse agonist at the CB2 receptor, contributing to its anti-inflammatory properties. Mengjie et al. demonstrated that CBD can act on CB2 receptors to reduce inflammatory responses, while also targeting TRPV1, G protein-coupled receptor 55 (GPR55), and 5-HT1A receptors independent of its cannabinoid-related actions. Specifically, CBD behaves as a 5-HT1A receptor agonist [40]. It enhances the effects of 8-OH-DPAT (a 5-HT1A receptor agonist) in promoting [35S]-GTPγS binding, indicating allosteric modulation of 5-HT1A receptors by CBD [41]. When applied topically, CBD also appears to lower oxidative and nitrosative stress [41]. A systematic literature review by David C. et al. identified 3–10 mg/kg as the optimal dosage range in animal models; however, their analysis did not include human clinical trials. As a result, further clinical studies are needed to establish the most appropriate CBD dose for dental applications. Although CBD’s anti-inflammatory and analgesic actions are well-recognized, the ideal therapeutic dose for consistent clinical outcomes in TMD (or orofacial pain) remains undetermined. Future investigations should focus on confirming both the safety and efficacy of CBD in managing TMD [42,43,44] (see Table 3, Table 4 and Table 5).

**Table 3:** Summary of CBD ways of administration—preclinical studies.

Reference	Results	Adverse Effects	Assessment Method	Model	Treatment Method
[45]	CBD reduced the increased orofacial allodynia and hyperalgesia in females	None	Von Frey and formalin tests	Animal (female rats, 276–322 g, n = 35)	Masseter muscle injections of CBD (10, 50, 100 µg in 10 mL)
[32]	CBD decreased mechanical sensitization and increased the mechanical threshold of masseter mechanoreceptors	None	Electronic Von Frey hair over the masseter muscle to measure the withdrawal response	Animal (female rats, (225–350 g, n = 54)	Intramuscular injection into masseter muscle – CBD (5 mg/mL) and CBN (1 mg/mL)
[29]	The reduction of the TMJ nociceptive behavior by III or III mGluRs agonists injected intracisternal together with sub-analgetic doses of WIN55,212-2 without causing cannabinoid-induced motor dysfunction.	Intra-articular administration of formalin produced scratches	Rotarod test	Animal (male rats, 220–280 g, n = 344)	The II or III metabotropic glutamate receptors (mGluRs) agonists alone or together with sub-analgetic doses of the WIN55,212-2 were injected intracisternal into formalin-inducted temporomandibular (TMJ) nociception

**Table 4:** Summary of CBD ways of administration—clinical studies.

Reference	Results	Adverse Effects	Assessment Method	Model	Treatment Method
[30]	The application of CBD formulation over masseter muscle reduced the masseter muscles activities and improved the myofascial pain condition	None	VAS scale EMG of masseter muscles assessment	Human (n = 60)	CBD skin application. Topical ointment was applied and rubbed gently over the skin surface (1.46% CBD, size of peas amount of formulation)
[34]	THC/CBD spray was beneficial for the majority of patients with peripheral neuropathic pain	dizziness (19%), nausea (9%), dry mouth (8%), dysgeusia (7%), fatigue (7%), somnolence (7%), and feeling drunk (6%)	NPS score, sleep quality 0–10 NRS score, intoxication 0–10 NRS score	Human (n = 380)	THC/CBD spray delivered 2.7 mg of THC and 2.5 mg of CBD to the oral mucosa

**Table 5:** Summary of modes of administration.

Study Type	Mode of Administration	Findings	Reference
**Preclinical Studies**	Intramuscular Injection (CBD alone, 5 mg/mL)	Reduced mechanical sensitization, increased mechanical threshold, effective for myofascial pain without neurological side effects.	[32]
	Electroacupuncture	Activated CB receptors, showing anti-inflammatory and antinociceptive effects in TMJ arthritis models.	[33]
	CBD Blocking NF-kB	Reduced interleukins, cytokines, chemokines, and pro-inflammatory growth factors, indicating anti-inflammatory properties.	[25,34,35]
	Intramuscular Injection (CBD in different doses)	Sex-dependent effects: Decreased orofacial allodynia and hyperalgesia, with better responses in male rats.	[36]
**Clinical Studies**	Transdermal Gel	Alleviated pain, cold sensations, and itching in patients with peripheral neuropathic pain.	[30,37]
	Topical Application (Over masseter muscle)	Reduced masseter muscle activity, improved masticatory muscle condition in TMD patients.	[30]
	Intramuscular Injection (CBD 5 mg/mL and CBD+CBN 1:1 mg/mL)	Effective for myofascial pain syndrome, no neurological side effects.	[38]
	Intraoral Application (Applied to mucosa)	Suggested as a potential route for CBD administration in orofacial pain relief.	[39]
	CB2 Receptor Activation	Inhibits inflammation, interacts with TRPV1, GPCR55, and 5-HT1A receptors, contributing to analgesic effects.	[40]
	Topical CBD Application	Reduced oxidative and nitrosative stress levels.	[41]
	Optimal Animal Model Dose	3–10 mg/kg; no established clinical dose for TMD yet.	[42,43,44]

The route of CBD administration is crucial for its application. CBD has mainly been used as a powder dissolved in saline for oral therapy. However, for the treatment of TMD, CBD should be used in another form, such as a gel or polymer film, to improve its absorption and reduce its rate of distribution. Based on a systematic review from 2022 that analysed 13 patients, CBD was used as a component of toothpaste, mouthwash solutions, gels, chewing gum, lozenges, and dental floss [42]. Although these forms of administration might be effective, they may not be as effective as a gel with long-term distribution.

Modern literature describes several analytical methods for determining cannabidiol concentrations, each offering distinct advantages in precision and application. High-Performance Liquid Chromatography (HPLC) is widely regarded as the gold standard due to its ability to accurately separate and quantify cannabinoids in various formulations [45,46,47]. Similarly, Gas Chromatography (GC) is a commonly used technique that provides comparable accuracy and precision to HPLC, making it a reliable option for cannabinoid analysis [46]. Another emerging method is Near-Infrared (NIR) Spectroscopy, which, when combined with partial least squares regression modeling, has been validated for quantifying CBD in liquid pharmaceutical formulations [47]. Additionally, Liquid Chromatography-Mass Spectrometry (LC-MS/MS) has been developed for assessing CBD and other cannabinoids in food matrices, ensuring robust and standardized analytical procedures. These methods play a crucial role in maintaining the quality and consistency of CBD products across various industries [45,46,47,48].

### 4.3. CBD as a New Therapeutic Strategy Reducing Other Symptoms of TMD

**Figure 3:**
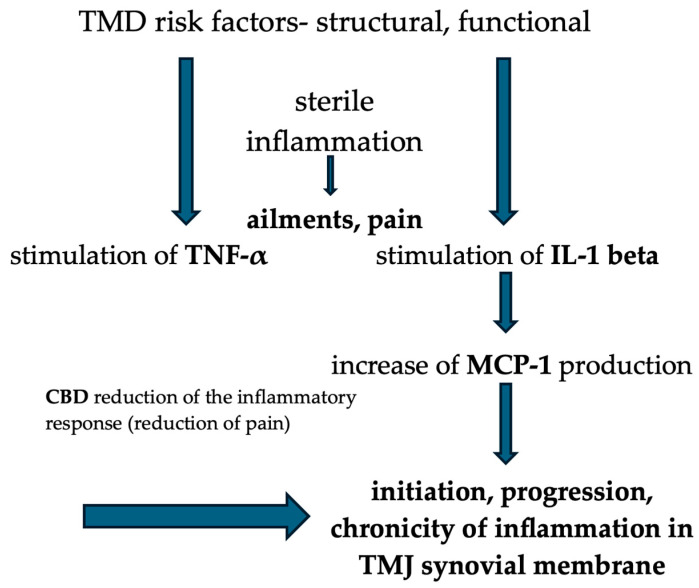
CBD influence on sterile TMJ inflammation.

Inflammatory pain refers to the heightened sensitivity to pain that spontaneously arises in response to tissue damage and/or inflammation [49]. In the context of temporomandibular joint (TMJ) pain, inflammation may contribute to its onset and various associated symptoms [50]. TMJ inflammation manifests as a sterile form of inflammation, akin to that observed in conditions such as atherosclerosis and Alzheimer’s disease [51]. Notably, a robust association exists between the concentration of pro-inflammatory cytokines in the synovial fluid and the level of pain experienced by patients in the TMJ area [49]. Individuals with internal derangement (ID) or osteoarthritis (OA) of the temporomandibular joint frequently exhibit symptoms of TMJ synovitis, which triggers the synthesis and release of pro-inflammatory cytokines from TMJ synoviocytes into the synovial fluid [50]. Among the most significant biomarkers of inflammation in TMJ synovial fluid are IL-1 beta and TNF-α [51]. Other inflammation biomarkers in temporomandibular disorders (TMD) include proteases, growth factors, and proteoglycans [49]. Studies have demonstrated a substantial correlation between cytokine concentration (IL-1 beta, TNF-α) and reported pain levels [50]. IL-1β and TNF-α stimulate the secretion of numerous biological factors into the TMJ synovial fluid, thereby significantly influencing the development of TMJ pain [51]. CBD inhibits or modulates the synthesis of cytokines, chemokines, and pro-inflammatory growth factors [52]. Pro-inflammatory cytokines such as TNF-α, IL-1β, IL-6, and Interferon-gamma are consistently reduced after therapy with CBD (Figure 3) [50]. Researchers have demonstrated that TNF-α increases the production of several chemokines, such as IL-8, CXCL1, and CCL20, in synovial fibroblasts from the TMJ [53]. TNF-α was initially known as a factor causing tumour necrosis, but it is now recognized as a major regulator of the inflammatory response [54]. It is produced by monocytes and macrophages and plays a crucial role in the inflammatory process by triggering numerous cytokines and chemokines [52]. TNF-α has been detected in the synovial fluid of patients with TMD and is involved in the destruction of the TMJ [50]. IL-1 beta is another well-known cytokine, besides TNF-α, that has been widely associated with pain and inflammation [53]. Some scientists have confirmed the involvement of this cytokine in the pathogenesis of temporomandibular disorders [54]. IL-1beta is considered a major proinflammatory biomarker involved in sterile inflammation [41]. TMJ inflammation represents the characteristics of sterile inflammation, so a new strategy of therapy may be based on applying CBD to reduce the concentration of cytokines, and consequently, reducing the level of reported pain [54]. Recently, a new mechanism of inflammation with the participation of IL-1 beta has been presented, where secretion of IL-1beta and IL-18, as well as activation of the inflammasome, which is a cytoplasmic protein complex, are involved in some diseases with sterile inflammation [46]. Stimulation of IL-1-beta in human TMJ synoviocytes causes an increased production of monocyte chemo-attractive protein-1 (MCP-1) [55]. MCP-1 is the main chemokine that induces migration of monocytes/macrophages and has a role in the initial stage of TMJ inflammation [56]. Other factors can also increase its levels, leading to subsequent progression and chronicity of inflammation in the TMJ synovial membrane [57]. When specific stimuli are sensed by cells, active caspase-1 triggers the transformation of IL-1 beta from its precursor to its mature form [58]. This transformation also leads to the transformation of IL-18 into its active form. As a result of this process, these interleukins are secreted outside the cells and trigger an inflammatory response [59,60,61]. In addition to IL-1beta and TNF-α, IL-6 also plays a crucial role in inflammation. IL-6 is another well-known proinflammatory cytokine that stimulates acute-phase proteins such as CRP. It plays a major role in chronic inflammatory diseases as well as cytokine storm [62]. Shinoda and Takaku have also demonstrated elevated levels of IL-6 and TIMP-1 (tissue inhibitor of metalloproteinase-1) in the TMJ aspirates of patients with non-inflammatory chronic temporomandibular joint (TMJ) disorders [63]. Interferon gamma is another inflammatory cytokine found in the synovial fluid of patients with TMJ disorder [61]. It is produced by T-lymphocytes and natural killer cells (NK) and has been found in synovial fluid along with TNF-α among patients with TMD. IFN-γ and TNF-α play a role in the development of pathological processes in TMJ structures. IFN-γ induces CXCR3 chemokines in the synovial fluid [62,63,64]. In a study conducted by Ohta K et al., the impact of IFN-γ in synovial fibroblasts obtained from the TMJ was examined. It was found that IFN-γ slightly increased the expression of CXCR3 chemokines in synovial fibroblasts obtained from the TMJ [62,63,64]. Moreover, IFN-γ together with TNF-α led to a dramatic increase in the expression of those chemokines [64]. The authors of this study suggested that the synergistic action of IFN-γ and TNF-α on the induction of CXCR3 chemokines may activate many T cells towards inflammation, which may lead to the pathological environment of the TMJ [64]. In summary, the pro-inflammatory cytokines discussed above can play a significant role in the development and progression of TMD. CBD has been studied for its potential to modulate the inflammatory response in various conditions, including TMD. The effect of CBD on pro-inflammatory cytokines is illustrated in Figure 3. The strong influence of CBD on IL-1β, IL-6, and IFN-γ levels is primarily attributed to its ability to modulate TLR signaling, inhibit NF-κB, influence CB2 receptor activity, activate PPAR-γ and A2A receptors, inhibit FAAH, and reduce oxidative stress. These mechanisms collectively suppress inflammatory cytokine production and contribute to CBD’s therapeutic potential in conditions characterized by excessive inflammation [65].

Among the many symptoms experienced by patients with temporomandibular disorder (TMD), poor sleep quality is one of the most frequently reported issues [66]. Additionally, obstructive sleep apnea (OSA) has been linked to various chronic pain disorders, including TMD [67,68,69]. This observed connection between sleep-disordered breathing and TMD underscores the need for further research to clarify the complex relationship between sleep and pain regulation [69]. In a study by Golanska et al. [1], the authors evaluated sleep quality and proposed that temporomandibular myofascial pain syndrome is shaped by the interplay of the limbic, autonomic, endocrine, somatic, nociceptive, and immune systems [1]. Given this complexity, monotherapy is often insufficient, prompting patients to seek alternative therapies such as cannabidiol (CBD) [1]. Indeed, additional studies have shown that 79.2% of patients with myofascial pain reported reduced anxiety, while 66.7% noted improved sleep after one month of CBD therapy [66,67,68]. Currently, occlusal splint therapy is the most employed treatment for TMD [69]. However, other options—such as physical therapy (including massages and Kinesio taping), behavioral modifications, platelet-rich plasma, botox and collagen injections, and pharmacotherapy—can be prescribed as adjuncts [70]. Various splint types alter occlusal conditions, condylar positioning, and vertical dimension, thereby modifying sensory input to the central nervous system and promoting muscle relaxation beyond just the masticatory muscles [71]. Consequently, an integrative, holistic approach is crucial in many TMD cases [69]. Nonetheless, clinicians often face challenges when TMD symptoms persist, prompting researchers to explore new methods to achieve muscle relaxation and reduce pain [70,71]. Cannabis, particularly CBD, is emerging as a potential therapeutic component in TMD management, with CBD offering a key adjunctive treatment that patients can use at home [69]. In dentistry, CBD can be delivered in various forms—including powder, solution, or gel—with evidence suggesting that it reduces masticatory muscle hyperactivity, inflammation, and contractions [70,71]. Despite its promise, questions remain about the optimal delivery method, concentration, and daily dosage, highlighting the need for continued research [71]. 

### 4.4. Limitations of the Evidence

A notable limitation of the current evidence on CBD for TMD is the overall scarcity of high-quality, controlled clinical trials, leading much of the available data to come from case studies, observational research, or preclinical models. Moreover, studies vary significantly in their methods of CBD administration—ranging from topical gels and injections to oral formulations—and in the dosages used, making direct comparisons problematic. There is also little consensus on how TMD outcomes, such as muscle hyperactivity and joint inflammation, should be measured or reported, limiting the ability to draw firm conclusions about efficacy and safety. Heterogeneity in patient characteristics (e.g., comorbidities, diagnostic criteria for TMD) further complicates data interpretation and reduces the generalizability of results. Finally, short follow-up periods and small sample sizes in many studies constrain insights into CBD’s long-term impact, underscoring the need for larger, well-designed randomized controlled trials that standardize treatment protocols and outcome assessments. Research on CBD primarily centers on its benefits for pain relief and anxiety, while its effects on lung function and the influence on smokers remain largely unexplored. The limited data available leave open questions about whether CBD might worsen or alleviate smoking-related lung irritation and inflammation, highlighting a critical need for further study in this area. 

Pharmacokinetic studies of cannabidiol (CBD) in humans reveal significant variability that is largely dependent on formulation and route of administration. Systematic reviews have shown that inhaled and oromucosal delivery result in relatively rapid absorption, with T_max_ typically occurring between 1 and 4 h, whereas oral formulations exhibit delayed T_max_ and low bioavailability—often as little as 6%—due to extensive first-pass metabolism [72]. Moreover, both C_max_ and the area under the curve (AUC) increase in a dose-dependent manner, with lipid-based formulations or administration in a fed state markedly enhancing systemic exposure [73,74,75]. For instance, smoking delivers a bioavailability of around 31%, although absolute bioavailability data for other non-inhalational routes remain sparse [73]. In addition, the elimination half-life of CBD varies with the route; while intravenous administration shows a half-life of approximately 24 h, chronic oral dosing can extend the half-life to 2–5 days. Despite these advances, substantial inter- and intra-subject variability persists, underscoring the need for further research to optimize dosing regimens across different patient populations [73,74,75].

### 4.5. Limitations of the Review Process

This review process faced several key constraints. First, only articles published in English or Polish were included, potentially omitting relevant studies in other languages. Second, by restricting the date range from 2007 to 2024, earlier research that might provide important foundational data on CBD and temporomandibular disorders could have been missed. Third, although multiple databases were searched, some relevant gray literature or unpublished data may not have been captured. Fourth, the initial screening of titles and abstracts was conducted by a single reviewer, which may introduce bias, even though disagreements were later resolved by a second reviewer. Lastly, inconsistencies in study designs, outcome measures, and reporting across the included articles limited direct comparisons and synthesis, potentially impacting the robustness of the conclusions drawn. 

### 4.6. Implications for Practice, Policy, and Future Research

The potential use of CBD as an adjunctive or standalone therapy for TMD opens multiple avenues for clinical practice and healthcare policymaking. Clinically, dentists and orofacial pain specialists may consider incorporating topical or oral CBD formulations into existing treatment regimens, such as splint therapy, physiotherapy, or pharmacotherapy, to address inflammation and muscle hyperactivity, although standard dosing guidelines remain unclear. At a policy level, clearer regulatory frameworks and guidelines for cannabis-based treatments in dentistry are needed to ensure patient safety and maintain consistent quality. Efforts to standardize CBD concentration, purity, and labeling would facilitate more reliable clinical applications. Looking ahead, future research should prioritize randomized controlled trials with larger sample sizes and uniform outcome measures to confirm efficacy, safety, and optimal dosing strategies. Additionally, studies exploring long-term effects, potential drug interactions, and variations among different formulations of CBD would further bolster the evidence base, guiding better clinical decision-making and policy development for TMD management.

## 5. Conclusions

Within the limitations of this review, current evidence suggests that cannabidiol (CBD) holds promise as a therapeutic adjunct for managing temporomandibular disorders (TMD). Multiple preclinical and preliminary clinical studies highlight that CBD may reduce muscle hyperactivity, alleviate inflammatory pain, and potentially improve patient-reported outcomes such as sleep and anxiety. These findings align with the review’s primary objective, which was to assess whether CBD could mitigate TMD symptoms and serve as a viable treatment option. Future research should also examine how CBD might best be integrated with conventional TMD therapies—such as occlusal splints, physical therapy, and behavioral interventions—to enhance patient outcomes. Continued high-quality investigations will clarify key factors such as the most effective administration route (e.g., transdermal gel, intraoral application, intramuscular injection), safe dosing ranges, and the long-term impact of CBD on TMD-related pain and function. As regulatory guidelines evolve, and more robust data become available, CBD-based interventions could play an increasingly important role in a multifaceted approach to TMD management.

## Figures and Tables

**Table 1:** Keywords used in the study.

Topic	Relevant MeSH Terms
Temporomandibular Disorders and Orofacial Pain	“Temporomandibular Joint Disorders” [MeSH]—“Temporomandibular Joint Dysfunction Syndrome” [MeSH]—“Myofascial Pain Syndromes” [MeSH]—“Orofacial Pain” [MeSH]—“Masticatory Muscles” [MeSH]
Cannabidiol and the Endocannabinoid System	“Cannabidiol” [MeSH]—“Cannabinoids” [MeSH]—“Cannabis” [MeSH]—“Endocannabinoids” [MeSH]
Pain and Inflammation	“Analgesics” [MeSH]—“Anti-Inflammatory Agents” [MeSH] “Inflammation” [MeSH]

**Table 2:** Selection criteria for papers included in this review.

Inclusion Criteria	Exclusion Criteria
1. Studies primarily investigating the role of CBD in managing TMD or related orofacial pain. 2. Clinical trials, observational studies, or case series reporting outcomes such as pain reduction, muscle hyperactivity, or joint function in TMD. 3. Systematic reviews, meta-analyses, or theoretical frameworks relevant to the endocannabinoid system’s role in TMD therapy or pain modulation. 4. Investigations examining CBD dosage, administration routes (e.g., topical, oral, intramuscular), or safety profiles in TMD contexts. 5. Articles published in English from 2007 to the present, ensuring up-to-date relevance.	1. Unrelated Topics: Studies not addressing TMD or lacking any focus on CBD interventions (e.g., no TMD outcomes or orofacial pain endpoints). 2. Editorials, commentaries, opinion pieces, or other gray literature without empirical or theoretical depth. 3. Studies that do not provide specific outcomes or mechanistic insights related to TMD or orofacial pain management. 4. Articles with missing data, abstracts only, or no full-text availability, preventing proper evaluation of methods/results. 5. Publications in languages other than English or those older than 2007, to maintain current applicability.

## Data Availability

Not applicable.
